# Do Fish Perceive Anaesthetics as Aversive?

**DOI:** 10.1371/journal.pone.0073773

**Published:** 2013-09-23

**Authors:** Gareth D. Readman, Stewart F. Owen, Joanna C. Murrell, Toby G. Knowles

**Affiliations:** 1 Brixham Environmental Laboratory, AstraZeneca, Freshwater Quarry, Brixham, Devon, United Kingdom; 2 School of Veterinary Science, University of Bristol, Langford House, Langford, North Somerset, United Kingdom; Université Pierre et Marie Curie, France

## Abstract

This study addresses a fundamental question in fish welfare: are the anaesthetics used for fish aversive? Despite years of routine general use of many agents, within both scientific research and aquaculture, there is a paucity of information regarding their tolerance and associated behavioural responses by fish. This study examined nine of the most commonly used fish anaesthetic agents, and performed preference tests using adult mixed sex zebrafish (*Danio rerio*), the most commonly held laboratory fish. Video tracking software quantified swimming behaviour related to aversion for each anaesthetic at 50% of its standard recommended dose compared with clean water in a flow-through chemotaxic choice chamber. Results suggest that several commonly used anaesthetics were aversive, including two of the most commonly recommended and used: MS222 (ethyl 3-aminobenzoate methanesulphate) and benzocaine. For ethical best practice, it is recommended that compounds that are aversive, even at low concentration, should no longer be used routinely for anaesthesia or indeed the first step of humane euthanasia of adult zebrafish. Two agents were found not to induce aversive behavioural responses: etomidate and 2,2,2 tribromoethanol. For the millions of adult zebrafish used in laboratories and breeding worldwide, etomidate appears best suited for future routine humane use.

## Introduction

The EU Directive [1] and its predecessor in the UK the Animals (Scientific Procedures) Act (A(SP)A) [2] place great emphasis on improving the welfare of those animals undergoing regulated procedures in scientific experiments via implementation of the principles of Reduction, Replacement and Refinement (the 3Rs). Similar legislation and ethical obligations exist in other regions of the world, but here we have chosen to concentrate on our UK experience for this study. Under the new EU directive [1] which updated A(SP)A [2] on the 1^st^ of January 2013, fish in scientific studies are protected under law from the point at which they become capable of independent feeding. Fish are increasingly used in experimental research for a variety of reasons, but not least, due to the high degree of genetic and physiological homology between fish and humans, and the drive to use alternatives to mammals in experimentation. The high fecundity, short lifecycles, small size and ease of culture of species such as zebrafish (*Danio rerio*) has resulted in very large numbers of these fish being held and used in laboratories. UK statistics from The Home Office (the relevant government regulatory body) for 2011 show that fish accounted for 563,903 procedures [3]. The majority of these fish were zebrafish. In addition to those within these official records, there were also significant numbers of zebrafish which are held for breeding purposes, supplying embryos for testing and those used before the point of protection under A(SP)A (and therefore not required to be reported). Globally it is estimated that in excess of 600 academic establishments are working with zebrafish [4].

Anaesthetic agents are routinely used for surgical procedures in fish (such as fin clips for genetic identification), but also as the first step of euthanasia for the majority of laboratory fish. Thus, most laboratory fish will experience anaesthetic agents at some point in their lives, and this likely equates to tens of millions per year globally. Humane induction of unconsciousness is a key characteristic of any agent used for anaesthesia or euthanasia, yet in the field of animal research very little has been done that considers the key elements of humane induction: initial perception and the potential for pain or distress associated with unconsciousness [5]. With regards to fish not only is there is a paucity of information regarding the aversiveness of anaesthetic agents to fish, there is little information on the actual capacity for these different drugs to ameliorate pain associated with surgery and other procedures. The UK body that addresses the ethical application of the A(SP)A legislation has specifically investigated this area and concluded that work was urgently required to assess the aversive nature of these agents for fish, especially when they are used as part of the process of euthanasia [6].

Anaesthesia, for fish, is an area where relatively little is known or formally reported, and has many practical difficulties. Further, the wide range of species and life histories makes generalisations across species extremely problematic. The number of drugs licensed for anaesthesia of fish is very limited and equipment and skills to monitor depth of fish anaesthesia are available to only a few specialist research groups globally. At present it is standard practice to monitor depth of anaesthesia based on visual assessment and interpretation of ventilation rates, posture or response to stimulus. This relies on the skill and experience of the scientist [7] but at present this approach is not based on any firm evidence. In most cases a degree of stress seems likely. Laboratory fish are generally group-housed to promote good welfare [8]. Therefore, an individual will need to be removed from its home tank and placed into an anaesthetic solution; or a chamber into which an anaesthetic agent can be added (equivalent to inhalation anaesthesia in mammals); or directly anaesthetised via injection (rarely done except for large fish species >10 kg). When anaesthetising fish it is common practice to place them into higher concentrations of anaesthetic solution for a quicker induction prior to moving them to a system that will maintain the required anaesthetic state [9]. This is not what would be considered best practice in other areas of animal research, for example when working with rodents, where a gradual induction of anaesthesia would be used to reduce any adverse effects on welfare [10] and/or a sedative administered prior to exposure to the anaesthetic.

MS222 (Ethyl 3-aminobenzoate methanesulfate) is likely the most common anaesthetic agent used for fish and is the only US Food and Drug Administration approved anaesthetic for use on fish destined for human consumption. It is registered for veterinary use with fish by Health Canada, and in the UK, Italy, Spain and Norway [7], [11], [12], [13], [14], [15]. MS222 appears to meet the five criteria for a suitable fish anaesthetic as suggested by Thienpoint and Niemegeers [16]. These are: high solubility in any matrix; high potency; complete recovery; ability for induction of a range of depths of anaesthesia. It also has a number of practical advantages, for example, as well as being soluble in a number of matrices (i.e. fresh and salt water) it can be administered via immersion or injection. Collectively these advantages make it suitable for use in a wide range of fish species in the laboratory, field or commercial aquaculture. Concerns, however, have been raised that MS222 may cause an aversive reaction in fish following exposure to the drug [17], and much anecdotal evidence exists as to the adverse reaction seen in fish during induction of anaesthesia [6]. One commonly used veterinary training text describes MS222 as an irritant [8], and in great detail another describes aversive reactions such as ‘coordinated excitatory behaviour with increased respiratory rate’ as part of an analgesia stage, leading to the next stage of ‘violent thrashing and jumping’ before eventually loosing equilibrium [19]. Gressler *et al*. [20] reported that MS222 caused a ‘detrimental physiological impact’.

Several papers have reviewed the physiological responses of fish to MS222 [7], [9], [14], [20], and one possible reason for behavioural responses occasionally reported [19] could be a reaction to the pH of the MS222 solution. Wedemeyer [21] reported that the use of unbuffered MS222 can result in longer term stress, caused by inter-renal ascorbic acid depletion. In order to reduce the pH stress, MS222 stock solutions are therefore routinely buffered to the pH of the holding tank water in the majority of UK laboratories, either by addition of NaOH or NaHCO_3_ solution, and then aerated. However, it has been reported that the efficacy of the MS222 can also be modulated using the pH, a factor often overlooked when administering the anaesthetic [9].

Despite MS222 being the ‘approved’ anaesthetic [9], others are commonly used in the research laboratories. Benzocaine is generally used for fish euthanasia as it causes rapid induction and is a fraction of the financial cost of MS222 (Table 1). Quinaldine is more commonly used for collecting fish in the wild and for surgical implantation of transponders in the field. Clove oil is used by some, possibly because of its ready availability to the public, the perception that it is a natural substance, and its low cost which means that larger fish, such as salmonid broodstock, can be safely and cheaply sedated for ease of collecting gametes. Other agents such as 2-phenoxyethanol (2-PE) are commonly used for routine anaesthesia in continental Europe, and increasingly in the UK. Further agents are reported in the literature (for a review see [22]), including 2,2,2 tribromoethanol (TBE), etomidate, isoeugenol, lidocaine hydrochloride, propoxate and quinaldine sulfate which we test here and also 4-styrylpyridine, piscine and propofol among others which are not so commonly used and were not tested in this study.

**Table 1:** Test substance effective dosage, identification and cost.

Test substance	Effective published dose*	Reference	Supplier	CAS N^o^	Cost £/litre of working solution
Hydrochloric acid (+ve control) pH 3.0	N/A	N/A	Sigma – Aldrich	7647-01-0	N/A
Ethanol 99.8% (solvent control)	1 ml/L	N/A	Sigma – Aldrich	64-17-5	N/A
TBE	4 mg/L	[49]	Sigma – Aldrich	75-80-9	£0.01
2-PE	0.3 ml/L	[50]	Sigma – Aldrich	122-99-6	£0.01
Benzocaine	100 mg/L	[26], [51]	Sigma – Aldrich	94-09-7	£0.08
Etomidate	2 mg/L	[28], [52], [53]	Ark Pharm Inc	33125-97-2	£0.09
Isoeugenol	20 mg/L	[54]	Sigma – Aldrich	97-54-1	£0.004
Lidocaine hydrochloride	100 mg/L	[55]	Sigma – Aldrich	6108-05-0	£0.02
MS222	100 mg/L	[9], [26], [56]	Sigma – Aldrich	886-86-2	£0.14
Propoxate	2 mg/L	[26]	Sigma – Aldrich	147-63-7	£0.24
Quinaldine sulphate	20 mg/L	[26]	Santa Cruz biotechnology	655-76-5	£0.08

* Effective dose  =  Dose at which Stage 5 anaesthesia is achieved. Where the referenced articles cite multiple alternative concentrations, the median was chosen. Costs were calculated in GBP and were correct at UK advertised prices in Nov 2012. They are included for a pragmatic comparison. AstraZeneca do not necessarily endorse or recommend any companies listed. Other suppliers were available and their costs may have been different.

Measures of initial aversion such as withdrawal, re-entry, dwelling time and increased activity have been used in several studies on both laboratory animals [4], [23], [24] and also farm animals [25]. These behaviours are chosen because they represent simple and objective measures that are easily quantifiable. The use of fish models for preference/avoidance testing is described in Pelkowski *et al*., [26]; percentage time spent in the untreated versus treated zones of water is used as a standard measure for calculating thresholds for aversive stimulus. This principle was used to test whether nine known fish anaesthetics, commonly used in both academia and industry (Table 1), were aversive when compared with untreated water. A positive control using Hydrochloric acid was also tested, as well as ethanol as a solvent control. Zebrafish were exposed to each anaesthetic in one half of a flow through chemotaxic tank, giving the opportunity for the individual to choose between anaesthetic or control environments. We hypothesised that fish would avoid the water containing hydrochloric acid and any other compound that it found aversive, and therefore choose to spend significantly more time in untreated water.

## Materials and Methods

### Ethics statement

This study was carried out under project and personnel licences granted by the Home Office under the United Kingdoms Animals (Scientific Procedures) Act [2], and also in accordance with AstraZeneca's local and global ethical policies.

### Test species and procedure

WIK strain mixed sex adult zebrafish (*Danio rerio*) were used from stock cultures. Greater detail of the fish and apparatus are given in the supplementary data file (File S1). Ten fish were subjected to each anaesthetic treatment, thus the study used 120 fish in total (10 fish×12 treatments).

Individual fish were transferred from stock tanks into the flow by means of a beaker containing a small volume of water. After the transfer, fish were allowed to acclimate for 150 seconds and subsequently a continuous dose of the test compound at a predetermined concentration was introduced into one of the mixing chambers, for a period of 150 seconds. The horizontal gradient created by the laminar flow within the tank allowed the untreated lane to remain uncontaminated, so creating two lanes between which the fish could move freely (Video S1). Following each experiment with one fish, the system was manually flushed to remove any test substance residues. The location and activity of the fish with access to both the treatment and untreated lane, were recorded via video camera for the whole experimental period. The video camera was positioned directly above the tank, ensuring that the camera did not create any shadowing on the water that could influence lane choice by the test fish. Offline analysis of the video recordings was carried out using VideoTrack analysis software (Version 2.5.0.25, ViewPoint, Lyon, France), and analysed over the 150 second exposure period; the results for each test substance were analysed separately. The data output from VideoTrack was subsequently formatted in Excel (Microsoft office, 2007) for statistical analysis using MLwiN [27] (available at http://www.bristol.ac.uk/cmm/software/mlwin/). The data were tested against a pre-specified, multilevel model. A multilevel approach was used as it allowed the data structure of the repeated measurements made on each fish (ie a measurement for each lane) to be taken into account. A general linear model within the multilevel model then included a term for the effect of treatment lane compared with control lane and also a term for the right hand side of the equipment compared with the left hand side, to ensure that no intrinsic bias was present within the experimental setup. These terms in the model were then tested, using a Chi square statistic, against a change in log likelihood. There was no evidence of a left/right flow chamber bias so only the parameter estimates of the effects of the anaesthetic treatments are presented here. Data deposited in the Dryad Repository: http://dx.doi.org/10.5061/dryad.8qv00.

### Test substance

Details of the anaesthetics examined in the experiment are listed in Table 1. Each was used at a concentration of 50% of the effective, published dose required to produce anaesthesia. All anaesthetic stocks were prepared in accordance with standard practice [22]. To achieve the high stock concentrations required for dosing, some compounds were first solubilised in ethanol (See File S1).

## Results

**Figure 1:**
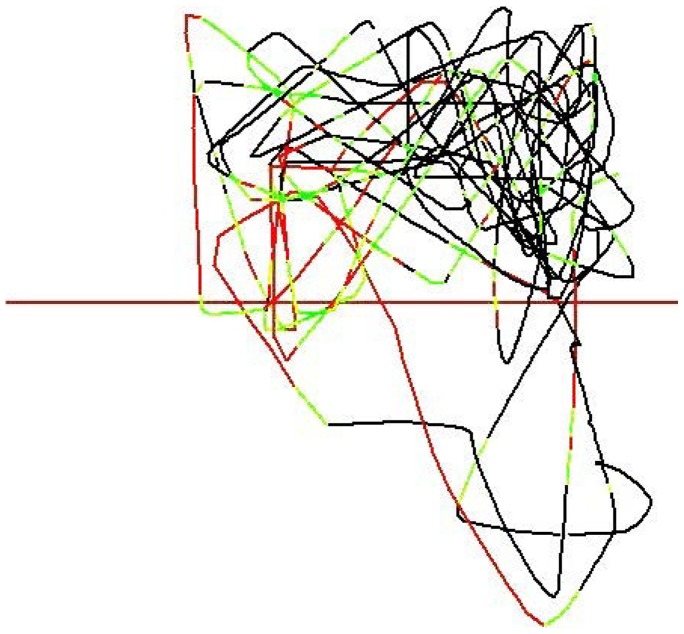
Image of the output from the ViewPoint software video tracked movement of a single adult zebrafish during exposure to hydrochloric acid (pH 3.0) viewed from above the choice chamber. The overlain red central line represents the point at which the two laminar flows meet. Hydrochloric acid is present in the lower lane of the image with the direction of flow right to left. Tracking indicates the aversive response to the acid area in the lower half, and preference for the dilution water of the upper lane. The tracking line is represented in different colours to indicate relative speeds of movement. Black representing slow, green moderate, and red fast swimming speeds.

No preference for the amount of time spent in either lane (right versus left) lane was demonstrated in the absence of test compounds, which demonstrates no environmental bias in the system. Hydrochloric acid was used as a positive control to assess the functionality of the system and to provide a model of aversive behavioural response. Of the nine anaesthetic substances tested, fish spent significantly less time in the exposure lane than the untreated lane in the presence of 2-PE, benzocaine, isoeugenol, lidocaine hydrochloride, MS222, propoxate and quinaldine sulphate (Table 2). These results strongly indicate that zebrafish experienced the substances as aversive. Differences in the time spent in the treated versus untreated lane, as a percentage of the total time, suggest it is possible to rank the compounds from the most to the least aversive (Figure 1), with quinaldine sulfate being the most aversive as test subjects spent the least time in the exposure lane, and lidocaine hydrochloride being the least aversive of those demonstrating a statistically significant effect. The parameter estimates produced by MLwiN (Table 2) show the average time spent in the control lane compared with the time spent in the exposure lane and any statistically significant difference. For example, if we look at the analysis for Time when looking at hydrochloric acid then the model shows that the time spent in the exposure lane was 62.5 seconds less than that of the control value of 102.7 seconds.

**Table 2:** Summary of MLwiN estimates (± se) of the effects for each of the anaesthetic agents in dilution water versus exposure for the response variables Time, Distance and Speed.

Candidate	Time in clean lane (secs)	Treatment lane difference (secs)	Distance in clean lane (mm)	Treatment lane difference (mm)	Speed in clean lane (^mm^/_sec_)	Treatment lane difference (^mm^/_sec_)
Control (No compound)	78.88 (4.375)	−8.26 (5.052)	6802.60 (561.894)	−231.76 (546.320)	86.65 (6.278)	4.98 (2.044)*
Ethanol (Solvent control)	68.40 (5.948)	−6.44 (6.868)	6462.77 (611.549)	−460.50 (504.659)	93.96 (6.210)	−0.23 (4.492)
Hydrochloric acid (Positive control)	102.75 (4.120)	−62.51 (4.757)***	7107.77 (565.213)	−3785.52 (501.593)***	74.57(7.704)	16.38 (7.110)*
TBE	79.33 (10.327)	−5.97 (11.925)	9378.96 (1684.200)	−948.76 (1400.031)	112.41 (21.932)	12.40 (18.142)
2-PE	78.15 (6.393)	−26.66 (7.382)*	6549.66 (821.706)	−1511.59 (937.802)	84.88 (6.557)	11.34 (2.828)***
Benzocaine	97.95 (6.078)	−35.77 (7.019)***	6691.17 (811.664)	−1796.39 (840.130)*	68.40 (8.504)	17.15 (4.797)***
Etomidate	84.22 (8.739)	5.97 (10.091)	7711.605 (850.185)	−788.55 (10.091)	93.78 (13.403)	−4.82 (9.422)
Isoeugenol	92.60 (3.239)	−42.91 (3.740)***	8721.39 (913.349)	−2860.26 (521.406)***	96.86 (11.367)	18.06 (1.990)***
Lidocaine hydrochloride	80.30 (8.467)	−20.29 (9.777)*	6637.70 (700.841)	−990.95 (702.988)*	89.99 (8.174)	11.28 (5.217)*
MS222	98.35 (13.845)	−36.79 (15.986)*	5994.61 (770.283)	−1635.58 (889.447)	65.59 (11.901)	2.23 (13.742)
Propoxate	89.63 (9.477)	−23.85 (10.943)*	8078.93 (1084.733)	−1191.47 (721.412)	84.24 (25.947)	18.09 (27.782)
Quinaldine sulfate	128.60 (4.620)	−94.97 (5.335)***	7077.03 (612.489)	−3626.36 (617.784)***	52.26 (8.201)	−53.12 (7.607)***

* P<0.05 **P<0.01 *** P<0.001.

The positive control, hydrochloric acid, induced a modification of normal swimming behaviour in that the distance swum in the acid was significantly shorter than that in the untreated lane in the same time period, showing an aversion to the acid in the exposure lane (Table 2). Benzocaine, isoeugenol and quinaldine sulphate also showed a statistically significant difference in the distance swum (mm) in the exposure versus untreated lane (Table 2) in that the distance swum in the control lane was greater than that in the exposure lane.

Average speed (mm/second) was also seen to increase significantly when fish were exposed to 2-PE, benzocaine, isoeugenol, lidocaine hydrochloride and quinaldine sulphate (Table 2) which further supports an aversive response to the test compounds. A significant difference was also seen in speed for the control exposure (dilution water only) in that fish moved on average faster in the left versus right lane, this result, however, was not strongly significant and was considered to be an anomalous result since there was no difference in time spent in either lane, nor distance travelled.

An additional solvent control was run using ethanol as the test substance, which showed no difference for any of the response variables (Table 2), suggesting that ethanol was not aversive at the level used in this study (0.033%). This lack of preference indicates that the use of ethanol as a carrier solvent does not create an aversive response in itself.

**Figure 2:**
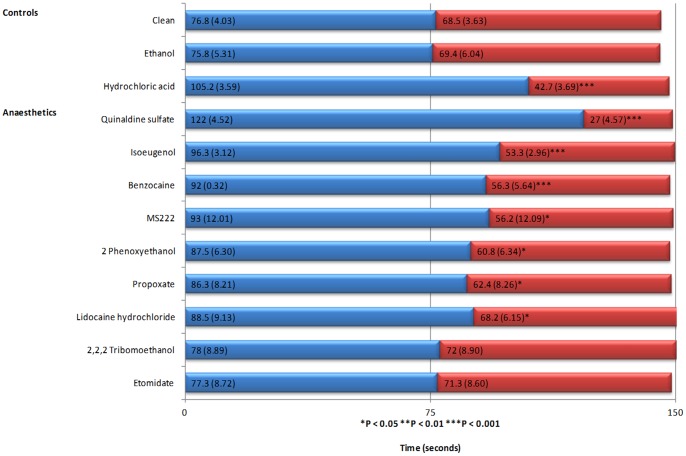
Shows the average time (seconds) spent in the exposure (red) and control (blue) lanes for each experiment (± SE, n = 10), ranked by aversion (highest to lowest) for the anaesthetics. N.B. Although each experimental run was 150

Figure 2 shows the typical VideoTrack output of a single fish during exposure to hydrochloric acid (pH 3.0) in one lane, demonstrating the aversive response of the fish to this compound. The extent of the tracking line within the untreated lane visually indicates that the aversion to hydrochloric acid caused the fish to spend more time in the untreated lane, also swimming a shorter distance and at a greater average speed in the acid.

Test subjects showed no difference with respect to any of the continuous response variables (Table 2) for the treated versus untreated lane for TBE and etomidate, which suggests that zebrafish do not find these test substances to be aversive.

## Discussion

The present study describes a novel chemotaxic preference test system for the quantitative assessment of commonly used anaesthetics for zebrafish. To the best of our knowledge there are no similar studies that assess behaviours indicative of aversion for fish during induction of anaesthesia. Of the nine anaesthetics assessed, seven elicited strong evidence of behavioural aversion. During exposure to these seven test substances, fish showed a statistically significant difference in the time spent in the exposure versus the untreated lane; additionally when fish were exposed to quinaldine sulfate, isoeugenol and benzocaine there was a significant difference in the distance travelled and average speed in the exposure versus the clean lane (Table 2) which leads us to rank their aversiveness (Figure 1).

Of the nine substances tested only etomidate and TBE showed no behavioural evidence of aversion. The safe use of etomidate has been investigated in fish [28], [29] and no negative allergenic, tissue irritation or blood chemistry response was observed in these studies. However, in a review of clinical anaesthesia in fish, Sneddon [7] reports that the use of metomidate hydrochloride, an etomidate derivative, has been shown to have negative effects in human and veterinary medicine. Beyond our intention to find a more humane method, we have not investigated potential long term effects in fish. Although the use of TBE did not elicit any aversive reaction it should be used with caution, as incorrect storage will result in the formation of potentially harmful degradation products [22].

The fact that MS222 elicited an aversive response in zebrafish at 50% of the effective dose is of great concern and supports the anecedotal evidence of its aversive nature put forward for salmonids [18], and other species [6], [17], [19]. Its continued use for this test species (*Danio rerio*) cannot be considered best practice especially as several other studies have reported a range of adverse physiological effects and differing stress responses to MS222 in several species [13], [30], [31]. Its use as a recommended anaesthetic for *Danio rerio* should therefore be revised and its use for other species re-evaluated. In addition, we provide new evidence to support a revision of the use of the other commonly used agents; benzocaine, 2-PE and isoeugenol. Millions of fish are exposed to these agents every year, and for zebrafish it appears there could be a more humane alternative.

Routinely in human and veterinary medicine, anaesthetic protocols are tailored to ensure a smooth transition (behaviorally and physiologically) to the desired state of anaesthesia (sedation, immobilisation, narcosis, amnesia). To our knowledge there are no similar studies that assess behaviours indicative of aversion for fish during induction of anaesthesia, despite there being a large body of literature on aversion of anaesthetics with respect to other laboratory animals. Several studies have shown altered physiological responses in fish during the induction of anaesthesia [9], [30], [31], but it is unclear whether the responses they describe are due to stress caused by aversion to the anaesthetic agent itself, or stress at the loss of the ability to control balance and orientation [30]. In other areas of veterinary medicine the use of multi-modal anaesthesia, where different drug combinations are used, with each component being responsible for achieving a different goal, is best practice to ensure the safest and most efficient induction possible [7], [31], [32]. If the induction of anaesthesia is likely to cause stress, either due to loss of control or aversion to the anaesthetic, then the use of pre-anaesthetic sedation in combination with an anaesthetic appropriate for the work to be undertaken is considered the humane method of choice [6]. This must also be the appropriate approach for use with fish. Indeed, it is now implied within the new EU directive [1] for all animals, including fish. However, there is as yet no other scientific evidence for fish that an appropriate protocol exists, or is justified. Here, our study offers the first behavioral evidence of an aversive response, and of most concern, an aversive response to the two agents that are the most commonly used and recommended, for the most commonly held fish species in the laboratory.

Using preference testing to assess any behavioural changes that can be associated with an adverse reaction to anaesthetics, at a level below that which would induce the initial stages of anaesthesia, allows the reaction to either a physical sensation (nociception, olfaction, taste) or behavioural (stress) caused by the exposure to be assessed. The use of this chemotaxic model allows a non-invasive, multifaceted approach with which it is possible to remove bias. Preference for physical position within the system was eliminated by making each lane uniformly similar, and further, any remaining intrinsic bias could be identified within the statistical analysis. The system was manually flushed after each exposure, removing any carryover of compound from the previous phase of exposure. The use of data generated by examining what happens in the untreated lane allows for each subject fish to become its own control, something which is extremely important due to the variation in behaviour within a population. Preference testing with fish is routinely carried out [26], [32], [33], [34], [35], and several assays using adult zebrafish [36], [37] and their larval forms [38], [39] are based on behavioural responses Zahl and co-workers [30] stated that substantial variations existed between the response of fish species to different anaesthetics and that large individual differences exist within each species. This varied response may be due to individual susceptibility of the test subjects to anaesthesia. Several studies have previously shown that individual fish have differing levels of susceptibility to anaesthesia [40], [41], [42], [43]. The reasons for these differences are unclear but may reside in individual variation, a combination of body size (and therefore relative respiratory rate and gill surface area), uptake kinetics and the presence of appropriate chemoreceptors.

Preliminary work looking at a positive control demonstrated that hydrochloric acid is aversive to zebrafish and that they display strong avoidance behaviour. The expression of altered behaviour has been shown to be an indicator of poor welfare in fish [44] and as such can be used as a negative behavioural measurement. The fact that the fish were able to detect the hydrochloric acid and show avoidance behaviour demonstrated that the system was able to elicit and detect a response and that it was suitable to be used in the assessment of the candidate anaesthetics. Previous workers have used direct application of acetic acid to elicit nociceptive responses [45], [46], [47], but here we have shown that zebrafish are capable of both detecting and behaviourally avoiding hydrochloric acid, and confirm very early work on olfaction [48].

Several of the compounds tested are either insoluble in water or were insoluble in water at the level required to create concentrated stocks for dosing into the laminar flows, and were therefore made up into solution using ethanol. The level of ethanol to which the fish were exposed was constant (0.033%) as preliminary work within this study on dosing had highlighted the fact that the use of different solvents and concentrations had the potential to disrupt the laminar flow. This was also the case for some of the compounds (TBE, 2-PE, benzocaine, etomidate and isoeugenol) and so a preliminary check was always carried out prior to each batch of experiments to ensure that the amount of disruption to the laminar flow if any, was acceptable, and would not affect the outcome of the study (see File S2). In the case of some potential test compounds the disruption was too great and these had to be rejected from the study (Acetic acid (positive control), clove oil). As the use of ethanol created an additional variable it was therefore important to run a solvent control in order to ensure that any differences in behavior seen were due to the test substance and not the solvent carrier. It is reasonable to say that the aversive effects seen in the exposures were solely due to the fish's reaction to the various anaesthetic agents themselves and not solely the ethanol carrier. However, it is a possibility that in some cases the ethanol/anaesthetic combination is aversive, but it was impractical to use the anaesthetic alone using this testing model.

It may be possible to look at long term preference testing models such as place preference, food reward and other cost benefit models to investigate the longer term effects of anaesthetic exposure and to also allow for the testing of anaesthetics which do not lend themselves to the model used in this study. This would be of benefit when looking at multiple exposures, such as with surgery.

The difference in behavioural reaction to the test compounds may in some instances be a result of the function of different chemoreceptor stimulation. It is unclear if the response to hydrochloric acid is driven by a similar response, such as olfaction or taste. Strieck [48] was able to show that acetic acid is detected by taste; by removing the forebrain of the Eurasian minnow (*Phoxinus phoxinus*) the fish's ability to discriminate substances by olfaction was removed, yet the test subjects were still able to detect and avoid the acetic acid. Hidaka [34] bisected the olfactory organs of Medaka (*Oryzias latipes*) to show an inability to detect certain aquatic contaminants by olfaction when compared with a non-bisected control. It may be possible to expose zeberafish bisected in this way to assess whether the difference in behavior is due to the route of detection. However, regardless of route it seems clear that zebrafish can detect and respond to several commonly used anaesthetics (quinaldine sulphate, isoeugenol, benzocaine, MS222, 2 PE, propoxate, lidocaine hydrochloride), but do not respond to others (TBE, etomidate).

## Conclusions

The results from this study show that zebrafish are aversive to the following anaesthetics commonly classified for use with fish: 2-PE; benzocaine; isoeugenol; lidocaine hydrochloride; MS222; propoxate and quinaldine sulphate. The aversive nature of these test substances established in this study suggest the preclusion of their routine use for adult zebrafish, and in future these compounds should perhaps be used only in exceptional circumstances. Zebrafish showed no aversion to TBE or etomidate. For future work with zebrafish the results reported here suggest the use of etomidate as a non-aversive alternative anaesthetic agents for adult zebrafish is likely to be preferable to that of TBE due to the reported potential issues that can arise with the latter caused by incorrect storage.

## Supporting Information

File S1 **Materials and Methods including a schematic diagram of the test apparatus.**
(DOCX)

File S2 **Photographic confirmation of maintenance of laminar flow during dosing.**
(DOCX)

Video S1 **Laminar flow in a chemotaxic chamber.**
(WMV)
