# Supplementary material for: Do Fish Perceive Anaesthetics as Aversive?
Source: PLoS One. 2013 Sep 23;8(9):e73773. doi: 10.1371/journal.pone.0073773 (PMC3781131; doi:10.1371/journal.pone.0073773)
Supplement: File S1 — Materials and Methods including a schematic diagram of the test apparatus. (DOCX) [file pone.0073773.s001.docx]

**File S1 – Materials and methods including a schematic diagram of the test apparatus**

**Materials and methods**

*Animals and housing*

Adult mixed sex Zebrafish (*Danio rerio*) WIK strain bred on site (Brixham Environmental Laboratory, AstraZeneca, Brixham, Devon, UK) and derived from an original stock obtained from the ZFIN centre (Eugene, Oregon) were used during this experiment. Stock fish held in a *Zebtec* aquatic housing system (Tecniplast, Italy), under a 14:10 light:dark photoperiod, at 28+ 1 ^o^C.

*Test apparatus*

The experimental apparatus consisted of a modified 45 litre (24”x12”x12”, length x width x height) glass tank (Clearseal, UK). The end panel was removed and a weir and coarse metal mesh (5mm) were added to prevent fish from escaping from the tank. A short segregation panel and fine mesh baffle (450 micron) were inserted at the other end of the tank to create two mixing chambers leading to the two lanes of water in a laminar flow, a second coarse mesh baffle was added to create an area of containment of equal length and width (Figure S1).

The flow rate of 3 L/min to each lane was regulated using variable flow ¾” valves (George Fischer, Ltd) with a rotameter (Blue-White Industries, Ltd F-400). The delivery pipe-work to each mixing chamber had an individual port to allow for the introduction of the test substance. Test substance delivery was via Watson Marlow Sci Q 400 peristaltic pumps. Each pump was calibrated by collecting the water output for a fixed length of time and determination of the delivered volume by weight. Calibration was carried out prior to each treatment to ensure the delivery of 3 ml/min or 1ml/min dependent on stock to be dosed. In order to achieve the required pH value for hydrochloric acid, the pH of the mixing chambers were monitored using Sartorius PB-11 pH meters and the dosing pumps were then adjusted so that sufficient acid was delivered to achieve the required pH.

**Figure S1**

**Flow regulator and rotameter**

**Test substance injection port**

**1^st^ baffle (5mm mesh)**

**2^nd^ baffle (450 micron mesh)**

**Position of laminar flow**

**Potential Anaesthetic dose**

**Water inlet Manifold**

Waste water weir

**Potential Anaesthetic dose**

**Partition wall**

**2^nd^ baffle (5mm mesh)**

To quantify the behavioural response of the fish to each treatment, the activity of the fish in the tank was captured on video from a fixed overhead position using a Toshiba Camileo H20 video camera. The digital recordings were then replayed for analysis using VideoTrack video analysis software (Version 2.5.0.25, ViewPoint, Lyon, France). The data output from VideoTrack was subsequently formatted for statistical analysis using MLwiN (Centre for multilevel modelling, University of Bristol).

*Dilution water*

The dilution water was dechlorinated tap water with salts added to maintain minimum hardness levels in accordance with Organisation for Economic Co-operation and Development (OECD) regulatory testing guidelines. The treated water was passed through a set of 20 and 10 µm filters and an ultraviolet steriliser before being delivered to holding tanks prior to distribution to the water system which delivered water in the laboratory at a temperature of 28 ± 1°C.

*Test Procedure*

Individual fish were transferred from stock tanks into the flow by means of a beaker containing a small volume of water, the beaker was submerged into the flow and the fish allowed to swim out. The fish were allowed to acclimate for a 150 second period to observe swimming performance and to allow a visible reduction in any erratic behaviour caused by the transfer. A continuous dose of each stock solution sufficient to create an anaesthetic dose 50% of the recommended working solution for full anaesthesia was then introduced into one of the mixing chambers for a period of 150 seconds. As a consequence of the horizontal gradient created by the laminar flow within the tank the un-dosed flow remained uncontaminated (Supplementary file S2). A new fish was used for each treatment to eliminate any bias caused by accumulation of previous test substance effects. The system was manually flushed following each experimental run in order to remove any residues from the previous experimental run. The overall functioning of the system was validated using Malachite green as a visual indicator (20mg/ml stock, dosed at 1ml/min) in order to assess the stability of the laminar flow and to also give a visual cue that the length of time calculated based on the flow:tank volume ratio was sufficient to completely flush the system and that there were no areas within the system were the construction had created eddies or pockets of slower moving water. During the initial method validation it was noted that different test substances, carrier solvents and volumes of solvent used affected the stability of the laminar flow and had the potential to create unwanted mixing. So as to ensure that each experimental run was comparable, the dosing of each candidate anaesthetic was assessed using Malachite green to track the stability of the laminar flow and were possible a pH indictor solution to check for any mixing (Fisher UK) (see supplementary dosing S3).

*Test substance*

The anaesthetics that were evaluated in the experiment are shown in Table 1. The concentrations were 50% of the effective published dose required to produce Stage 5 anaesthesia according to the available literature. All anaesthetic stocks were prepared in accordance with standard practice [1]. In some cases due to the high concentration of anaesthetic stock required to achieve the correct working concentration after injection into the appropriate flow certain test substances were completely solubilised in 50 ml of 99.8% ethanol (Sigma-Aldrich) (Table S1). Due to the low pH of quinaldine sulphate the stock was buffered using sodium bicarbonate (Sigma-Aldrich) to a pH 5.0 in order to maintain a neutral pH during the experimental runs.

**Table S1**

| Test substance | Effective published dose* | Reference | Supplier | Stock base |
| --- | --- | --- | --- | --- |
| Hydrochloric acid pH3.0 (positive control) | N/A | N/A | Sigma - Aldrich | N/A |
| Ethanol 99.8% (solvent control) | 1ml/L | N/A | Sigma - Aldrich | N/A |
| 2,2,2 Tribomoethanol | 4 mg/L | [2] | Sigma - Aldrich | Water |
| 2-Phenoxyethanol | 0.3 ml/L | [3] | Sigma - Aldrich | Ethanol |
| Benzocaine | 100 mg/L | [1,4] | Sigma - Aldrich | Ethanol |
| Etomidate | 2 mg/L | [5,6,7] | Ark Pharm Inc | Ethanol |
| Isoeugenol | 20 mg/L | [8] | Sigma - Aldrich | Ethanol |
| Lidocaine hydrochloride | 100 mg/L | [9] | Sigma - Aldrich | Water |
| MS222 | 100 mg/L | [1,10,11] | Sigma - Aldrich | Water |
| Propoxate | 2 mg/ml | [1] | Sigma - Aldrich | Water |
| Quinaldine sulphate | 20 mg/L | [1] | Santa Cruz biotechnology | Water |

*Effective dose = Dose at which Stage 5 anaesthesia is achieved. Where the referenced articles cite multiple alternative concentrations, the median was chosen.

*Data Analysis*

The digital video recordings were transferred via SD card to a Desktop computer (Dell Optiplex GX520) and replayed for analysis. Analysis of the video from each treatment using Video Track allowed for the tracking of movement within customised areas as defined by the user in terms of time spent within these areas the distance travelled and entry counts into each area. Following the automated analysis a spreadsheet was produced by Video Track software detailing time, distance and movement within the predetermined areas at intervals of 30 seconds for the duration of each treatment. The relevant sections during the time periods where subjects were exposed to anaesthetic concentrations were then exported into an Excel spreadsheet, this data was then formatted for input into the statistics package MLwiN.

**References**

1. Ross, L.G. Ross, B. Ross, B. 2008. *Anaesthetic and Sedative Techniques for Aquatic Animals*. Wiley-Blackwell, Oxford.
2. McFarland, W. Klontz, G. 1969. Anesthesia in fishes. Federation proceedings. **28**. 1535-1540.
3. Velisek, J. Wlasow, T. Gomulka, P. Svobodova, Z. Novotny, L. 2007. Effects of 2-phenoxyethanol anaesthesia on sheatfish (Silurus glanis L.). *Vet. Med-Czech*. **52**. 103-110.
4. Weber III, E.S. 2011. Fish Analgesia: Pain, Stress, Fear Aversion, or Nociception? *Vet. Clin. N. Am*. **14**. 21-32.
5. Amend, DF. Goven, BA. Elliot, DG. 1982. Etomidate: effective dosages for a new fish anesthetic. *T. Am. Fish. Soc*. **111**. 337-341.
6. Limsuwan, C. Limsuwan, T. Grizzle, J.M. & Plumb, JA. 1983. Stress response and blood characteristics of channel catfish (Ictalurus punctatus) after anesthesia with etomidate. *Can. J. Fish. Aquat. Sci*. **40**. 2105-2112.
7. Plumb, J. Schwedler, T., Limsuwan, C. 1983, Experimental anesthesia of three species of freshwater fish with etomidate. *Prog. Fish. Cult*. **45**. 30-33.
8. Preperation of anaesthetic baths. Technical note series 3. [www.aqui-s.com](http://www.aqui-s.com) (accessed 28 November 2012)
9. Houston, A. Czerwinski, C. Woods, R. 1973. Cardiovascular-respiratory activity during recovery from anesthesia and surgery in brook trout (Salvelinus fontinalis) and carp (Cyprinus carpio). *J. Fish. Res. Board Can*. **30**. 1705-1712.
10. Küçük, S. 2010. Efficacy of tricaine on Peocilia latipinna at different temperatures and concentrations. *Afr. J. Biotechnol*. **9**. 755-759.
11. Carter, KM. Woodley, CM. Brown, RS. 2011. A review of tricaine methanesulfonate for anesthesia of fish. *Rev. Fish. Biol. Fisher.* **21**, 51-59.
