# Supplementary material for: Do Fish Perceive Anaesthetics as Aversive?
Source: PLoS One. 2013 Sep 23;8(9):e73773. doi: 10.1371/journal.pone.0073773 (PMC3781131; doi:10.1371/journal.pone.0073773)
Supplement: File S2 — Photographic confirmation of maintenance of laminar flow during dosing. (DOCX) [file pone.0073773.s002.docx]

**File S3 – Photographic confirmation of maintenance of laminar flow during dosing.**

Images show the stability of the laminar flow during dosing. Each compound is dosed with Malachite green as an indicator so as to follow the progression of the compound. Flow is always left to right and the dye and compound are dosed in the same lane.

**Figure S2**

**
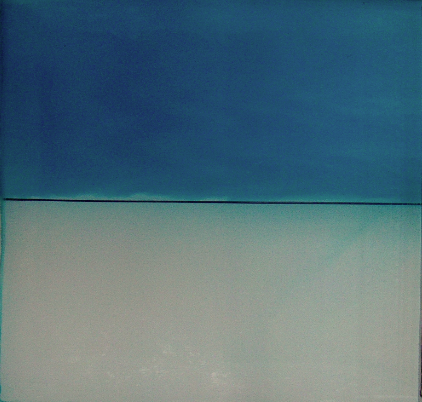
**

No test substance in the system

**Figure S3**

**
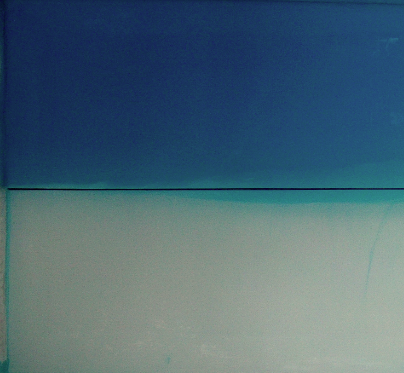
**

Hydrochloric acid in the Top lane

**Figure S4**

**
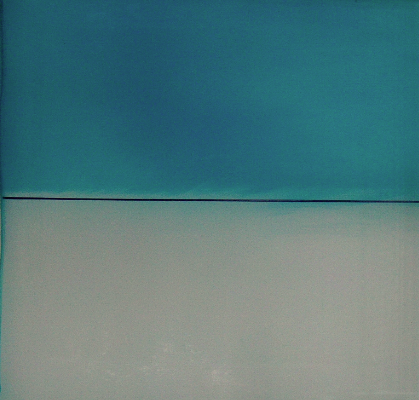
**

Ethanol in the Top lane

**Figure S5**

**
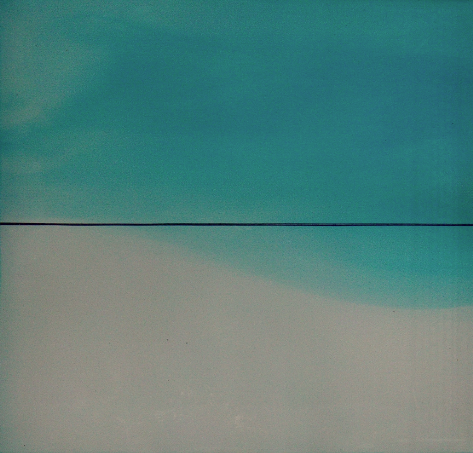
**

2,2,2 Tribromoethanol in the Top lane

**Figure S6**

**
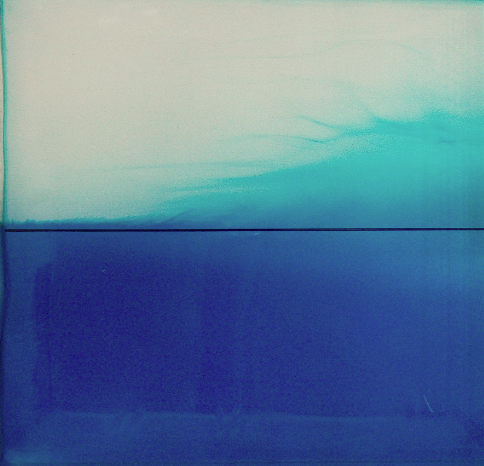
**

2-Phenoxyethanol in the Bottom lane

**Figure S7**

**
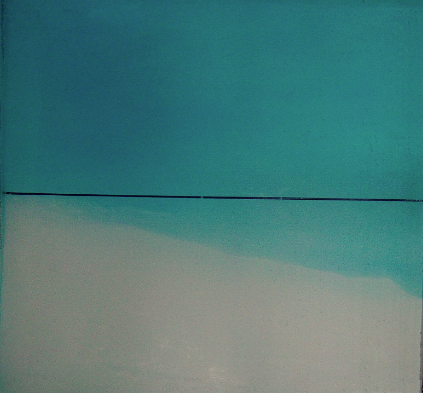
**

Benzocaine in the Top lane

**Figure S8**

**
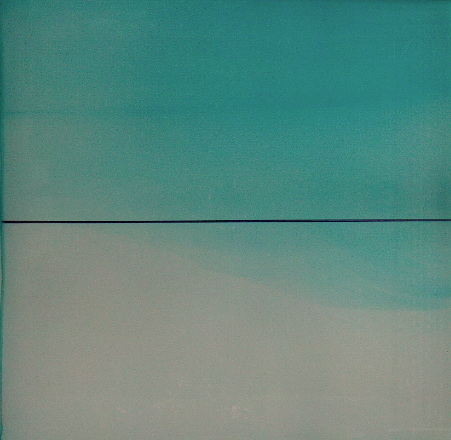
**

Ethomidate in the Top lane.

**Figure S9**

**
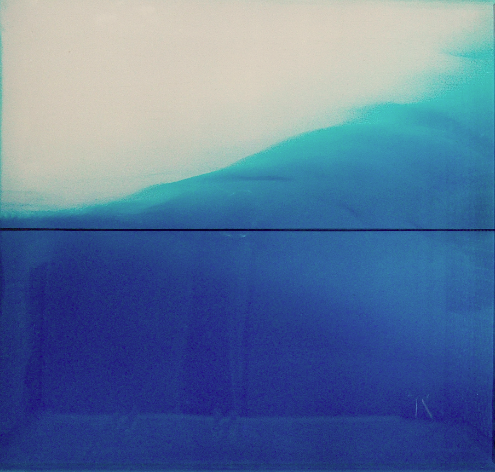
**

Isougenol in the Top lane.

**Figure S10**

**
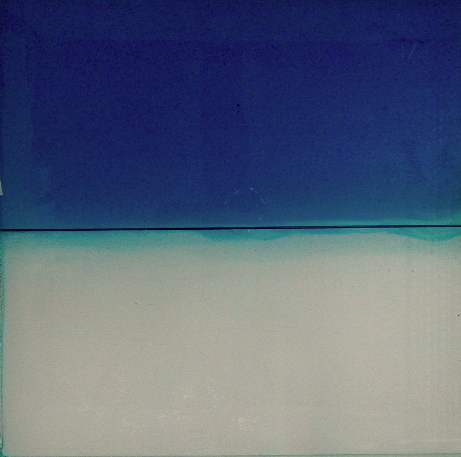
**

Lidocaine hydrochloride in the Bottom lane.

**Figure S11**

**
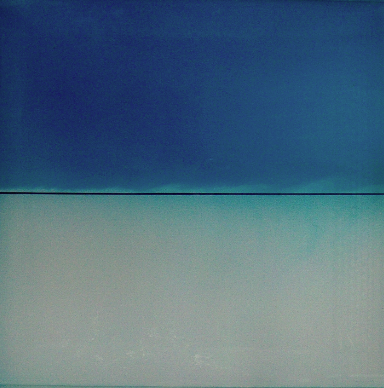
**

MS222 in the Top lane.

**Figure S12**


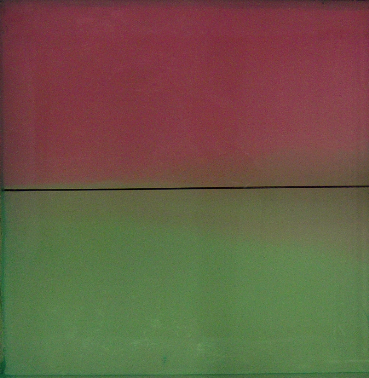


MS222 in Top lane with Universal indicator solution pH3-10 (Fluka).

**Figure 13**

**
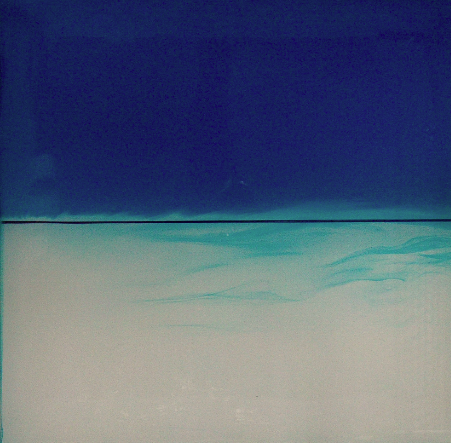
**

Propoxate in the Top lane.

**Figure 14**

**
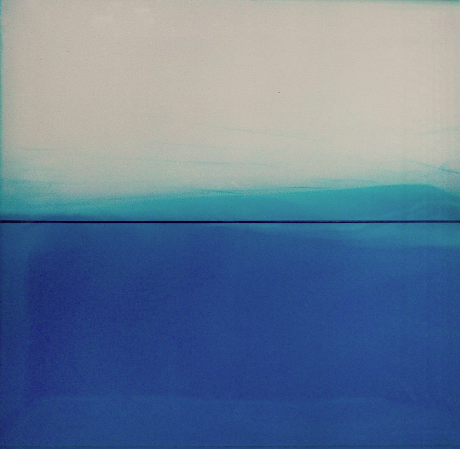
**

Quinaldine sulphate in the Bottom lane.
